# Can you trust this source? Advice taking in borderline personality disorder

**DOI:** 10.1007/s00406-022-01539-w

**Published:** 2023-01-11

**Authors:** Jakob Scheunemann, Lena Jelinek, Sarah V. Biedermann, Michael Lipp, Amir H. Yassari, Simone Kühn, Jürgen Gallinat, Steffen Moritz

**Affiliations:** 1 Department of Psychiatry and Psychotherapy, University Medical Center Hamburg-Eppendorf, Hamburg, Germany; 2 Lise Meitner Group for Environmental Neuroscience, Max Planck Institute for Human Development, Berlin, Germany

**Keywords:** Advice taking, Decision-making, Judge-Advisor System, Trust bias, Psychotic-like cognitive bias, Social influence

## Abstract

Research suggests that patients with borderline personality disorder (BPD) share a range of cognitive biases with patients with psychosis. As the disorder often manifests in dysfunctional social interactions, we assumed associated reasoning styles would be exaggerated in a social setting. For the present study, we applied the Judge-Advisor System by asking participants to provide initial estimates of a person’s age and presumed hostility based on a portrait photo. Afterwards, we presented additional cues/advice in the form of responses by anonymous previous respondents. Participants could revise their estimate, seek additional advice, or make a decision. Contrary to our preregistered hypothesis, patients with BPD (*n* = 38) performed similarly to healthy controls (*n* = 30). Patients sought the same number of pieces of advice, were equally confident, and used advice in similar ways to revise their estimates. Thus, patients with BPD did trust advice. However, patients gave higher hostility ratings to the portrayed persons. In conclusion, patients with BPD showed no cognitive biases in seeking, evaluating, and integrating socially provided information. While the study implies emotional rather than cognitive biases in the disorder, cognitive biases may still prove to be useful treatment targets in order to encourage delaying and reflecting on extreme emotional responses in social interactions.

**Supplementary Information:**

The online version contains supplementary material available at 10.1007/s00406-022-01539-w.

## Introduction

Borderline personality disorder (BPD) is a mental disorder that, according to the DSM-5 [1], has severe symptoms such as suicidal [2, 3] and non-suicidal self-harm behavior [4, 5] as well as impulsive behaviors [6, 7]. Different theories on BPD highlight patients’ abnormalities in terms of how patients process information, draw conclusions, and make sense of the world, especially in the social domain (for a review of theories, see [8]). Understanding the cognitive underpinnings of BPD may help to develop and improve cognitive [9, 10] and metacognitive interventions [11].

BPD patients show a range of reasoning biases [12], such as deficits in problem solving/planning [13–15] and decision making [16]. Additionally, comorbidity in patients with BPD is high (especially with depression [17] and PTSD [18]) which may promote additional cognitive biases and cognitive dysfunction [19, 20]. Patients with BPD also self-report elevated levels of jumping to conclusions (JTC) and belief inflexibility [21, 22], which are so-called psychotic-like cognitive biases as they were originally linked to psychosis [23]. JTC, for example, is observed not just in full-blown psychosis but also in remitted psychosis [23], in healthy participants with elevated levels of psychotic-like experiences [24], and even in relatives of patients with psychosis [25, 26]. A linkage of psychosis-like reasoning styles in patients with BPD is compatible with studies reporting that up to 60% of patients with BPD display psychotic symptoms [27–29]. However, there is a lack of experimental studies investigating JTC and belief inflexibility in BPD (with only one exception [30]). Of particular interest are reasoning styles within a social setting as BPD core symptoms usually occur in social contexts. For example, the use of information from a social source may be influenced by a trust bias [31, 32]. Our goal in this study was thus to investigate reasoning and decision making in a social context by applying the Judge-Advisor System to patients with BPD.

### Applying the Judge-Advisor System to borderline personality disorder

The Judge-Advisor System (JAS; [33]) has mostly been used in social and organizational psychology (for a review, see [34], for a meta-analysis, see [35]) but has also been adapted for research on psychosis [36] and depression [37].

The JAS paradigm consists of a judgment and an advice phase. In the judgment phase, participants make an initial judgment, e.g., estimating the age of a person based on a photograph. In the advice phase, participants receive advice, e.g., in the form of responses by previous respondents. Participants are free to adjust or not to adjust their initial judgment. The main outcome is the degree to which each participant changes their initial judgment in response to advice. Studies with clinical populations found that patients with schizophrenia [36] and depression [37] adjusted their initial estimates more than controls after having received advice. Our group [38] developed the paradigm further; after participants received the first advice, they could either give a final estimate right away or could seek further advice before giving a final estimate. In line with the JTC bias [24], participants with more frequent psychotic-like experiences sought less advice before making a final decision than participants with an average frequency of psychotic-like experiences [38].

The JAS has been demonstrated to be a valuable tool for investigating reasoning and decision making in the social context, specifically how advice is sought and used. For the present study, we are the first to apply the JAS to BPD. Participants made judgments about persons based on portrait photographs of the persons. One such judgment was neutral (estimating the person’s age), and the other judgment was related to BPD symptomology (rating the person’s hostility). BPD patients tend to rate others as, for example, less trustworthy [39–41]. After receiving advice in the form of (fabricated) answers of previous respondents, participants could seek additional pieces of advice before making their final decision.

Applying the JAS may inform us about how patients with BPD seek and use advice. This has high practical relevance (e.g., patients with BPD have an increased risk of discharge against medical advice; [42]) and advances our knowledge of cognitive processes in psychiatric disorders (e.g., for a review on recent advancements in predictive processing on the way patients use information to update beliefs, see [43]).

### Hypotheses

The JAS paradigm provides measures on information sampling (number of pieces of advice before making a decision), confidence in the decision, and information integration/belief flexibility (adjustment of estimate in response to advice). For each of the three outcomes, we formulated a specific hypothesis (see below).

#### Information sampling

Preliminary evidence suggests that patients with BPD seek less information before coming to a conclusion. First, patients “jump to conclusions” according to self-report [21, 22]. Second, patients with BPD more often show a JTC bias than healthy controls on the experimental beads task. In this probabilistic reasoning task, patients with BPD sample less information before concluding which of two jars (with different ratios of beads) the instructor is drawing beads from [30]. Also, patients with BPD make riskier (e.g., [44]) and more impulsive decisions [45] than controls. A possible JTC bias might be elevated if the information provided is of a social nature (e.g., in the form of advice from previous respondents), as patients with BPD show a trust bias [31], feel more readily excluded [46], and show increased rejection sensitivity [47, 48]. Furthermore, JTC may represent a transdiagnostic trait, including suicidality and depressiveness [49], both of which patients with BPD are highly prone to [2, 50]. In sum, we assumed that patients with BPD would show a hasty decision-making style (JTC) and thus would seek less advice than controls before giving their final estimate.

H1: Patients with BPD seek less advice than controls.

#### Confidence

The JAS can also investigate confidence in judgments. Two studies suggest an overconfidence bias for patients with BPD; that is, patients with BPD are more often highly confident in judging emotions when such confidence is not justified [51] or show too high confidence in false memories [52]. Thus, this presumed overconfidence bias should lead to increased confidence in final judgments by patients with BPD compared to controls.

H2: Patients with BPD rate their confidence higher than controls.

#### Belief flexibility

Advice weighting is the degree the estimates are adjusted in line with advice, which is a measure of belief flexibility. Patients with schizophrenia and participants with elevated levels of psychotic-like-experiences use advice more [36, 38]. As a follow-up study showed, this increased weighting of new information may be explained by aberrant processing of information like hypersalience towards the newly provided advice/information [53]. One can assume similar aberrant processing of information in patients with BPD. Work from computational psychiatry using associative learning tasks suggests that patients with BPD show similar processes to patients with schizophrenia [54], especially in response to social compared to non-social cues [55]. Hence, we expected increased advice weighting among patients with BPD.

H3: Patients with BPD weight advice more than controls.

This study is the first to test the JAS paradigm in patients with BPD. The (adapted) JAS task allows to investigate how patients with BPD evaluate and use socially provided information. As outlined above, we assumed patients with BPD (compared to healthy controls) to seek less advice, to be more confident and to weight advice more.

## Methods

### Preregistration and ethics

On 20 June 2018 (time-stamped), we non-publicly uploaded our study protocol on AsPredicted (#12037; https://aspredicted.org/xv9fn.pdf). At that point, data from four patients (10.5%) and six healthy controls (20%) had already been collected but not analyzed. Prior to data collection, the local medical board’s ethics committee approved the trial (trial #PV5263).

### Participants and recruitment

We recruited *n* = 38 in-patients with BPD via the University Medical Center Hamburg-Eppendorf as well as *n* = 30 healthy controls (HC) via leaflets and word of mouth. Inclusion criteria for patients and healthy controls were (a) age 18–65 years, (b) fluency in German, (c) IQ ≥ 70 as estimated by a vocabulary test, and (d) no neurological disorder. An additional criterion for the clinical sample was a BPD diagnosis according to the DSM-IV, verified with the German version of the Structured Clinical Interview for DSM-IV (SCID-II, [56]). The exclusion criterion was a previous psychotic episode, tested via the Mini International Neuropsychiatric Interview (M.I.N.I., [57]). We verified mental health status in controls with the M.I.N.I.; none of the healthy controls reported having previously suffered from any psychological disorder or having sought psychotherapeutic or psychiatric treatment. Samples did not differ according to years of age (BPD: *M* = 25.7 (*SD* = 10.6), HC: *M* = 27.8 (*SD* = 7.4), *t* (65.23) = 0.95, *p* = 0.344) or gender (BPD: 84.2% female, HC: 80.0% female, *χ*^2^(1) = 0.205, *p* = 0.651).

### Experimental condition

#### Estimation task

**Fig. 1:**
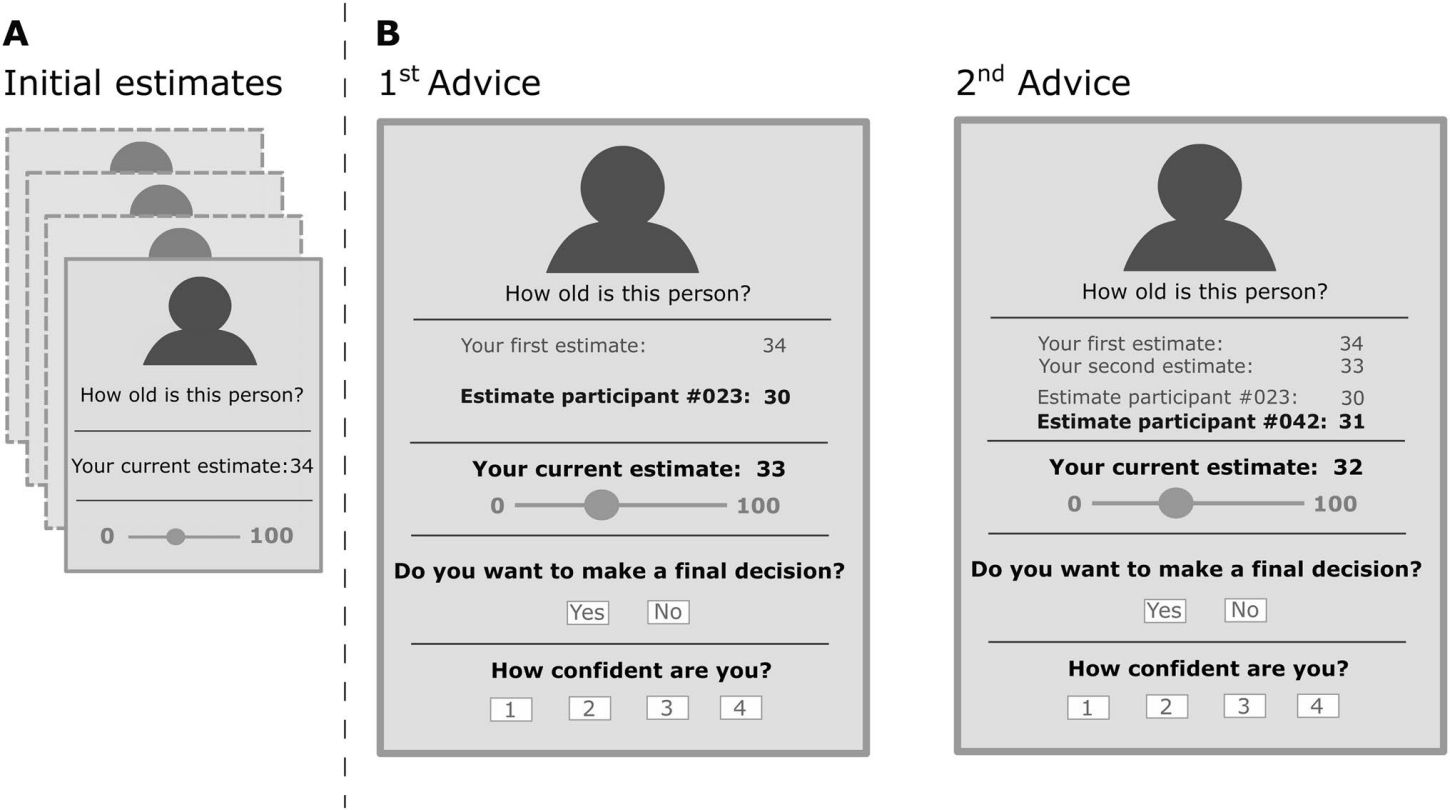
Schematic representation of the Judge-Advisor System (see methods for exact wordings). **A** Participants consecutively saw portrait pictures of four different persons, for which they each gave initial estimates (age and hostility). **B** In the second step, participants saw the same portrait pictures in the same order along with advice, which we framed in the experiment as “estimates from previous participants.” Participants could give a new, possibly revised estimate. Additionally, participants could decide whether they want to make a final decision or see additional advice (minimum of one piece of advice, maximum of four pieces of advice per trial). Further, they rated confidence on a four-point scale. At all points, participants saw the portrait picture, all their previous estimates, and all previous pieces of advice. In the example above, the participant’s initial age estimate was 34 (**A**). In the advice phase (**B**), the participant saw the (fabricated) previous answer by participant #023, who estimated the person as 30 years old. The participant adjusted their estimate to 33 in response to the advice. After having seen the second advice (“Estimate participant #042: 31”), the participant adjusted their estimate to 32

The procedure followed the standard sequence of a Judge-Advisor System (JAS; [34]), illustrated in Fig. 1. Participants made judgments about four White people (ages 23, 25, 32, and 43; order randomized across participants) based on a portrait photo (displayed with 450 × 338 pixels) taken from the Siblings Database of the CG&V Group [58]. First, participants answered the question “How old is this person?” (*Age task*) for all four portraits in random order, followed by the question “How hostile is this person?” (*Hostility task*) with the photos in the same order. Participants entered their estimates on a visual analogue scale (using a slider) with a range from 0 (labeled “not hostile”) to 100 (labeled “hostile”). After participants gave their initial estimates, we showed them the same photos, this time along with advice. In all instructions, we avoided the term “advice.” Instead, the instructions said that they would see randomly selected answers from participants in a pilot study (e.g., “Estimate from participant #99: 45). However, this advice was not real; we computed it, based on each participant’s initial estimates, in the same way for all participants (see Table 1). Advice distances were larger for the Hostility task than for the Age task, as we expected estimating someone’s hostility to be more difficult and thus less precise than estimating someone’s age (as an indication, standard deviations were more than five times larger on the initial hostility than on the initial age estimates, see below). Participants had the opportunity to change the position of the slider and decide whether they wanted to see more answers of previous participants or make a final decision without seeking further advice. After a maximum of four pieces of advice, the trial terminated. In all instances, participants saw all previous estimates and all previous pieces of advice. In each step, participants also rated their confidence on a 4-point scale (1 = *unconfident*, 2 = *rather unconfident*, 3 = *rather confident*, and 4 = *100% confident*).

**Table 1:** Advice in relation to the initial estimate

Sequence	Age Task				Hostility Task			
	a	b	c	d	a	b	c	d
1st Advice	+ 2	− 4	− 6	+ 8	+ 5	± 15	± 30	± 45
2nd Advice	+ 1	− 3	− 5	+ 10	− 7	± 7	± 20	± 58
3rd Advice	+ 3	− 5	− 8	+ 7	− 2	± 20	± 32	± 38
4th Advice	+ 2	− 4	− 5	+ 7	+ 4	± 18	± 38	± 39

The order within each sequence (a–d) was fixed, but the allocation to the trial (1–4) was randomized across participants. For sequences b–d for the Hostility task, the advice was in the negative direction for initial estimates > 50 and in the positive direction for initial estimates ≤ 50. The mean advice of all four pieces of advice per sequence equaled the first advice for each sequence, except sequence a in the Hostility task (which equaled 0). Advice was truncated to 0 or 100 for computed advice that resulted in a number < 0 or > 100, respectively. For example, an initial hostility rating of 55 would have resulted in advice for sequence d of 10, 0, 17, and 16, in that fixed order

#### Scoring

We calculated all scores according to our study protocol. The *Number of Requests for Advice* (NoRfA) was the number of times a participant requested advice before giving the final estimate (range 0–3), and *Confidence* was the participant’s confidence rating for the final estimate (range 1–4). As the standard measure for advice integration [34], we calculated *Relative Averaged Advice Weighting* (RAAW) by dividing the difference between the final and the initial estimate by the difference between the advice and the first estimate (RAAW = [final estimate—initial estimate]/[advice—initial estimate]). When there were multiple pieces of advice, the advice was averaged [59]. In line with our study protocol and previous studies (e.g., [60]), we truncated relative advice-taking scores > 1 to 1 and scores < 0 to 0. We averaged all scores on the subject level separately for both task types (age and hostility) and calculated pooled scores over both tasks.

### Psychopathology and procedure

The study design was cross-sectional. Prior to the in-person assessment, participants completed the German version of the 23 item Borderline Symptom List (BSL-23, [61]), the 12-item Rosenberg Self-Esteem Scale, the Beck Depression Inventory-II (BDI, [62]), and the Beck Scale for Suicidal Ideation (19 item version; BSS [63, 64]). During the approximately 2-h long assessment, participants completed the JAS paradigm and the vocabulary test as a proxy for IQ as well as other unrelated experiments as part of a larger study. Further, a trained and supervised rater conducted the M.I.N.I. and rated participants’ depression with the 17-item version of the Hamilton Depression Rating Scale (HDRS; [65]). At a second approximately half-hour long in-person assessment in the following days, an again trained and supervised rater conducted the SCID-II interview for BPD. For their participation in the entire study, each participant received a reimbursement of 20 Euros.

### Preregistered analysis and deviations

We preregistered a sample size of 40 patients with BPD and 40 healthy controls, determined by budget. For two patients, the JAS paradigm was not conducted as the assessments had to be terminated early (resulting *n* = 38). As this study was part of a larger study, not all healthy controls were included to avoid group differences in age and gender, resulting in *n* = 30. This decision was made prior to data analysis.

In line with our preregistration, we calculated group differences for the outcome measures NoRfA, Confidence, and RAAW with Welch’s *t* tests separately for both tasks (age and hostility) as well as for the pooled scores across both tasks. Even though it was not specified in the study protocol, we also report the effect size measure Cohen’s *d* (|*d|*< 0.2 negligible, |*d|*< 0.5 small, |*d|*< 0.8 medium, |*d|*≥ 0.8 large) and their corresponding 95% confidence intervals, both calculated with the R package *effsize*. The mediation analysis—linear relationship between symptom severity (BSL-23) and advice weighting (RAAW), *not* mediated by self-esteem (RSS)—was calculated according to Baron and Kenny [66], as specified in the preregistration. We calculated the Pearson correlation as correlation coefficients (*r*), although this was not explicitly specified in the study protocol. We did all analyses using R Studio 1.1.456 [67, 68].

## Results

Patients showed severe symptomology (see Tables 2). Borderline symptomology was higher (*M* = 56.4) than in the BPD validation sample (47.2; [61]). 32 of the 38 patients (84%) with BPD had a BDI-II score of at least 29, indicating severe depression; the remaining 6 patients (16%) had scores indicating moderate depression [62]. According to the M.I.N.I., the great majority of patients (89.5%) had a lifetime depressive disorder, with 28 out of 38 patients (73.7%) currently fulfilling criteria for a depression. About half of the patients had an alcohol-use disorder (55.3%) and/or any non-alcohol substance-use disorder (39.5%); for other disorders according to the M.I.N.I. interview, see Table S1 in the supplementary materials. Compared to controls, patients also had much higher scores on self-reported suicidality (BSS) and depression (HDRS) as well as much lower scores on self-reported self-esteem (RSES). 31 patients were prescribed psychopharmacological treatment at the time of the assessment (81.6%), with an additional four (10.5%) having received psychopharmacological treatment not currently but previously; the remaining three patients (7.9%) had never been medicated.

**Table 2:** Group differences in psychopathology

	BPD (*n* = 38)	HC (*n* = 30)	*t* test^a^	*d*	95%-*CI* *d*
BSL-23	56.4 (17.0)	3.8 (6.7)	*t*(50.49) = 17.49	3.91	[3.09, 4.74]
BDI-II	37.4 (8.3)	2.8 (4.1)	*t*(56.61) = 22.54	5.12	[4.12, 6.12]
HDRS	20.2 (5.1)	0.8 (1.6)	*t*(45.43) = 22.13	4.89	[3.92, 5.86]
BSS	16.1 (8.4)	0.0 (0.2)	*t*(37.04) = 11.81	2.56	[1.9, 3.2]
RSES	18.1 (4.4)	36.1 (4.5)	*t*(62.09) = 16.50	4.03	[3.19, 4.88]

*CI* Confidence interval. *BSL-23* Borderline symptom list (23-item version). *BDI-II* Beck depression inventory-II. *HDRS* Hamilton depression rating scale. *BSS* Beck scale for suicidal ideation (19 item version). *RSES* Rosenberg self-esteem scale

^a^All group differences *p* < 0.001

### Judge-Advisor System

All initial age estimates were in the range 24–62 years. Patients and healthy controls estimated with similar accuracy the age of the depicted persons (mean absolute deviation BPD: *M* = 6.9 (*SD* = 3.3), HC: *M* = 7.4 (*SD* = 3.3); *t*(61.57) = 0.66, *p* = 0.513; *d* = 0.16, 95% *CI* [− 0.33, 0.65]). Patients rated the persons as more hostile than healthy controls with medium effect size (mean initial hostility rating: BDP: *M* = 37.8 (*SD* = 19.1), HC: *M* = 23.3 (*SD* = 17.6); *t*(64.36) = 3.25, *p* = 0.002; *d* = 0.79, 95% *CI* [0.28, 1.29]).

### Preregistered results

There were no significant group differences in any preregistered outcome (see Table 3). In comparison to healthy controls, patients with BPD requested negligible (and non-significantly) more advice; rated their confidence slightly (but non-significantly) lower with small effect size; and weighted advice slightly (but non-significantly) stronger with small effect size. This pattern was the same for the Age task and the Hostility task as well as for the pooled average across both tasks. Thus, results did not confirm any of the preregistered main hypotheses.

**Table 3:** Means and group differences on the main outcomes of the JAS task

	BPD (*n* = 38)	HC (*n* = 30)	*t* test	*p*	*d*	95%-*CI* *d*
NoRfA						
Pooled score	0.55 (*SD* = 0.60)	0.46 (*SD* = 0.64)	*t*(60.29) = 0.57	0.572	0.14	[− 0.35, 0.63]
Age task	0.59 (*SD* = 0.69)	0.50 (*SD* = 0.70)	*t*(61.77) = 0.54	0.589	0.13	[− 0.36, 0.62]
Hostility task	0.51 (*SD* = 0.68)	0.43 (*SD* = 0.62)	*t*(64.56) = 0.52	0.607	0.12	[− 0.36, 0.61]
Confidence						
Pooled score	2.57 (*SD* = 0.52)	2.71 (*SD* = 0.47)	*t*(64.83) = − 1.19	0.240	− 0.29	[− 0.78, 0.20]
Age task	2.55 (*SD* = 0.53)	2.67 (*SD* = 0.47)	*t*(65.11) = − 0.99	0.326	− 0.24	[− 0.73, 0.25]
Hostility task	2.59 (*SD* = 0.61)	2.75 (*SD* = 0.55)	*t*(65.01) = − 1.17	0.247	− 0.28	[− 0.77, 0.21]
RAAW						
Pooled score	0.18 (*SD* = 0.17)	0.13 (*SD* = 0.10)	*t*(62.35) = 1.40	0.168	0.32	[− 0.17, 0.81]
Age task	0.22 (*SD* = 0.20)	0.17 (*SD* = 0.15)	*t*(65.71) = 1.20	0.234	0.28	[− 0.21, 0.77]
Hostility task	0.13 (*SD* = 0.16)	0.09 (*SD* = 0.11)	*t*(64.19) = 1.20	0.235	0.28	[− 0.21, 0.77]

*CI* Confidence interval

### Post hoc analysis of main outcomes

As outlined in the supplementary materials S1, we ran a series of post hoc analysis. However, there was no indication for an effect of certain trial types (different distances of advice) and no indication that outliers contributed to the incorrect conclusion of the null-effects. Furthermore, currently depressed patients with BPD in our sample (*n* = 28, according to the M.I.N.I.) did not significantly differ from healthy controls on any pooled outcome (NoRfA: *d* = 0.06, *p* = 0.824; Confidence: *d* =  − 0.34, *p* = 0.208; RAAW: *d* = 0.27, *p* = 0.316), objecting to a previous finding in which participants with depression had higher advice-taking scores than healthy controls in a JAS task [37].

### Correlational analyses

We preregistered an expected relationship between symptom severity (BSL-23) and advice weighting in the patient group. However, there was no indication of a correlation between these two variables (*r* =  − 0.02, *p* = 0.898, *n* = 38). Additionally, symptom severity was neither correlated with NoRfA (*r* =  − 0.08, *p* = 0.612, *n* = 38) nor with Confidence (*r* = 0.06, *p* = 0.738, *n* = 38).

Moreover, no pooled score (NoRfA; Confidence, RAAW) correlated significantly with any other investigated variable in the patient sample (*n* = 38): Rosenberg Self-Esteem Scale (*r* ≤  − 0.31, *p* ≥ 0.057), Beck Depression Inventory-II (*r* ≤ 0.10, *p* ≥ 0.545), HDRS (*r* ≤ 0.21, *p* ≥ 0.215), Beck Suicidal Scale (*r* ≤ 0.14, *p* ≥ 0.417), or estimated IQ (*r* ≤|0.26|, *p* ≥ 0.11). Consequently, and further elaborated in the Supplementary Materials S1, there was no indication of a mediation by self-esteem on the relationship between symptom severity and JAS outcome measures. See Supplementary Materials S1 for correlations between outcomes.

## Discussion

In this study, we introduced the Judge-Advisor System (JAS; [33]) to investigate the extent to which patients with BPD seek and use advice. In this task, participants first estimated the age and presumed hostility of a person based on the person’s portrait photo. Second, they received advice in the form of (fabricated) estimates by previous respondents. Participants could subsequently adjust their initial estimate and decide whether to seek additional advice or not. We assumed that patients with BPD would seek less advice, would use advice less to adjust their estimates, and would be more confident in their final estimates compared to healthy controls. Our hypotheses were largely unconfirmed. No significant group differences were found. Hence, the JAS did not reveal any deficits in patients with BPD in their ability to process, seek, or integrate advice by anonymous previous participants.

Our study and hypotheses were prompted by prior research that has linked BPD with cognitive biases initially found in patients with schizophrenia [21, 22], which we assumed would be rather elevated as the information was provided in a social context in which patients with BPD tend to have difficulties. It is thus surprising that patients with BPD did not show deficits on the JAS paradigm, a well-grounded paradigm from social and organizational psychology [34] that has provoked deviant behavior in clinical populations diagnosed with depression [37] and psychosis [36]. In contrast, patients with BPD even performed somewhat better than controls by showing higher accuracy in their initial age estimates, sought somewhat more advice before making a decision, adjusted their estimate slightly more strongly in the direction of the advice (which is generally considered more optimal; [59]), and were less confident (which could be considered functional in view of high ambiguity).

The findings were robust across different experimental manipulations. Results were similar for both the neutral task type (estimating a person’s age) and the borderline-specific task (estimating a person’s hostility). Different distances of advice (difference between advice and initial estimate) did not evoke any group differences. Also, post hoc analysis did not suggest that results were driven by outliers. Moreover, there were no correlations with psychopathological measures on borderline symptomology, depression, or suicidality. Finally, the subgroup of patients with BPD currently fulfilling criteria for depression did not differ from healthy controls (contradicting a previous study by Hofheinz [37]).

In conclusion, our hypotheses were not confirmed. In the context of advice, patients with BPD thus seemed able to use other participants’ advice to improve their answers in an estimation task. In the light of the vast research on the trust bias in BPD [31], it is worth noting that our data do not suggest any primary cognitive bias hampering patients with BPD from correctly evaluating and integrating information coming from other participants.

First, patients did not show a jumping to conclusions bias (JTC bias; [69]) despite the symptom overlap with psychosis [27] and previous indications of a JTC bias in patients with BPD [22, 30]. Even though previous studies using this paradigm did show a JTC bias in psychosis-prone individuals [38], other studies failed to replicate the JTC bias in patients with schizophrenia with novel [70] or even classic paradigms [71]. While some studies have suggested the JTC bias as a transdiagnostic trait [49], evidence from this paradigm does not support this idea. However, concurrent validity between this novel JAS paradigm and classical JTC tasks needs to be further established (for initial evidence, see [38]), especially as healthy controls also sampled a smaller total number of information in the JAS paradigm (i.e. smaller NoRfA) compared to the typically observed total number of information on other JTC tasks, for example the beads task (i.e., Draws to Decision).

Second, regarding confidence, patients with BPD were slightly—but non-significantly—less confident than healthy controls, which again counters one of our hypotheses. Findings thus speak against an overconfidence bias in BPD, which we assumed based on previous findings [51, 52]. On the other hand, results also do not indicate reduced confidence of patients with BPD compared to healthy controls, which was found in other studies (e.g., confidence in judging emotions of others; [72, 73]). Hence, more research is needed to disentangle factors influencing state confidence in patients with BPD. Our study did, however, replicate lower trait self-esteem in patients with BPD compared to healthy controls [74]. However, self-esteem did not correlate with the number of pieces of advice taken or the participant’s confidence and correlated only weakly (but non-significantly) with advice taking. This goes against a previous study in individuals with depression [37] and research from student samples [75].

Third, although we had assumed that patients with BPD would weight advice more than healthy controls, no group differences emerged; patients adjusted their advice marginally more than controls. Hence, in term of belief updating, the data suggest neither a belief inflexibility (previously suggested by Puri et al. [22]) nor an overadjustment/aberrant belief updating (previously suggested by Henco et al. [54]). Instead, the process of belief updating in BPD remains complex [43].

In sum, patients with BPD sought and used anonymous advice the same as healthy controls. This highlights that patients with BPD do not seem to have cognitive biases in evaluating information coming from others. Patients with BPD were not overly hasty and did not naively use information from others (i.e., overadjusting, increased confidence), nor were they overly skeptical (i.e., seeking or using less advice). Instead, patients evaluated their own and others’ skills in the estimation tasks in a healthy manner. In other words, patients with BPD trusted the previous participants’ judgements. Thus, this study also contributes to the vast research on trust in BPD (for a recent review on the trust bias in BPD, see [31]): Interestingly, patients with BPD rated the persons depicted in the photos in the estimation task as more hostile than healthy controls (which is in line with [76, 77]), indicating less trust by patients in others a priori; however, patients trusted the anonymous advice neither more nor less than controls, indicating no diminished trust in the socially provided information. To conclude, the distrust of patients with BPD was not ubiquitous.

To integrate the findings with other research on trust in BPD, we have to note one important aspect of the JAS paradigm used here: participants were given no reason to expect hostile advice as it was given in the form of “randomly selected answers by previous participants.” This means that patients trusted the anonymous advice of a stranger, who had no knowledge about the existence of the patient and who could be expected to answer the estimation question to the best of their knowledge. In that sense, the paradigm resembles studies with little interaction with the trustee. For example, Preuss et al. [78] did not find decreased trust in a one-round trust game in a non-social risk condition in which participants were playing with a computer that was randomly selecting from the actual behavior of previous participants. That study resembles the selection of advice in our study (also “randomly selected”). Thus, it may be less a deficit in trusting others per se (“trust bias”) that drives patient’s behavior and more the fear that a particular individual might exploit or reject the patient. In that sense, patients with BPD might rather show an “untrustworthiness sensitivity” [79], in which they would have a stronger reaction to and expectation of untrustworthy behavior than healthy controls. Whether this reaction is actually deficient per se or, in light of the life history of patients with BPD, even an adaptive behavior stands to debate [80]. Most importantly, results from this study point to the need to be careful when assuming distrust in patients with BPD.

We would like to draw several cautious inferences from the data. Patients with BPD attribute more hostility to others, but this emotional bias is not general; for example, they are not less open to advice than healthy individuals, suggesting that patients can trust others, such as therapists, in some situations. Also, patients did not require additional information or interaction with or about the advisors, suggesting that in anonymous therapeutic settings like online interventions, patients might be open to advice. To disentangle this, future studies should manipulate information about the advisor (e.g., an expert giving advice) and see whether the finding translates to therapeutic content.

This study also adds to the field of cognitive bias research on BPD. For schizophrenia, basic research has identified specific cognitive biases that are successfully targeted in interventions, such as metacognitive training [81]. Tentative evidence from metacognitive training adapted for patients with BPD (B-MCT) shows that modifying biases may indeed slow decision-making and ameliorate symptoms [11]. In light of the present findings, however, the magnitude of cognitive distortions seems to be weaker in BPD than in schizophrenia. Thus, the B-MCT’s modules on JTC and memory/overconfidence should be reconsidered critically. Yet, while a hasty decision making style might not be a core BPD feature, patients with BPD might still benefit from improved metacognitive skills to counter frequent over-reactions and one-sided judgments typically seen in the disorder. This study suggests that metacognitive interventions can draw on patients’ high metacognitive capacity to help patients to access these skills more readily in highly arousing, real-life situations.

The study has some strengths, in our opinion. The analyses were preregistered, which by some experts is regarded as an important criterion for good scientific practice [82], and the range of manipulations of the JAS paradigm speaks for the robustness of the detected results. However, as in most clinical studies, a clinical comparison group is missing and sample sizes were small. A larger sample would have possibly allowed to investigate whether certain biases are associated with specific BPD features not present in all patients. While patients and controls did not differ on the core sociodemographic variables age and gender, subsample sizes were not balanced. Possibly, our recruitment was somewhat biased as we included only currently hospitalized patients who are help-seeking and thus may be more open to seeking advice than non-hospitalized patients. Previous or current treatment could have also influenced patients’ behavior (however, we tried to included participants as shortly as possible after admission). Also, psychiatric symptomology in our patient sample was above average, which goes along with factors that may exert an effect on cognitive biases like psychopharmacological treatment [83, 84], comorbidity, and psychosocial factors like lower socioeconomic status [85]. Future studies should manipulate aspects of the given advice more rigorously, such as by showing multiple pieces of advice and providing more information about the advisor. Other adjustments to the paradigm could be to use other estimates than age or to change the scales (e.g. using probability estimates as a confidence scale). Future studies might also want to consider race in the study design and analyses.

## Conclusion

Applying the JAS paradigm from social psychology, we investigated how patients with BPD seek and use (socially provided) information. In two estimation tasks, patients with BPD did not behave differently compared to healthy controls. Patients sought the same number of pieces of advice before submitting their final estimate, were similarly confident in their final estimate, and adjusted their initial estimate equally in response to the advice. This contradicted our preregistered hypotheses. These results suggest that the lack of trust in others observed in patients with BPD may not be driven by cognitive biases but rather by other factors such as emotional biases (e.g., rejection sensitivity). This raises the hope that patients with BPD may be able to deal with advice the same way healthy individuals do, for example, when receiving advice within a therapeutic setting or in systemic therapy approaches like family therapy.

## Supplementary Information

Below is the link to the electronic supplementary material.

Supplementary file1 (DOCX 21 KB)

Supplementary file2 (DOCX 13 KB)

## References

[1] American Psychiatric Association DS, Association AP (2013). Diagnostic and statistical manual of mental disorders: DSM-5.

[2] Black DW, Blum N, Pfohl B, Hale N (2004). Suicidal behavior in borderline personality disorder: Prevalence, risk factors, prediction, and prevention. J Pers Disord.

[3] Paris J (2019). Suicidality in borderline personality disorder. Medicina (B Aires).

[4] Zanarini MC, Frankenburg FR, Reich DB, Fitzmaurice G, Weinberg I, Gunderson JG (2008). The 10-year course of physically self-destructive acts reported by borderline patients and axis II comparison subjects. Acta Psychiatr Scand.

[5] Reichl C, Kaess M (2021). Self-harm in the context of borderline personality disorder. Curr Opin Psychol.

[6] Weiner L, Perroud N, Weibel S (2019). Attention deficit hyperactivity disorder and borderline personality disorder in adults: a review of their links and risks. Neuropsychiatr Dis Treat.

[7] Berg JM, Latzman RD, Bliwise NG, Lilienfeld SO (2015). Parsing the heterogeneity of impulsivity: a meta-analytic review of the behavioral implications of the UPPS for psychopathology. Psychol Assess.

[8] Gunderson JG, Fruzzetti A, Unruh B, Choi-Kain L (2018). Competing theories of borderline personality disorder. J Pers Disord.

[9] Cristea IA, Gentili C, Cotet CD, Palomba D, Barbui C, Cuijpers P (2017). Efficacy of psychotherapies for borderline personality disorder: a systematic review and meta-analysis. JAMA Psychiat.

[10] Storebø OJ (2020). Psychological therapies for people with borderline personality disorder. Cochrane Database Syst Rev.

[11] Schilling L, Moritz S, Kriston L, Krieger M, Nagel M (2018). Efficacy of metacognitive training for patients with borderline personality disorder: Preliminary results. Psychiatry Res.

[12] Koudys JW, Gulamani T, Ruocco AC (2018). Borderline personality disorder: refinements in phenotypic and cognitive profiling. Curr Behav Neurosci Reports.

[13] Ruocco AC (2005). The neuropsychology of borderline personality disorder: a meta-analysis and review. Psychiatry Res.

[14] Abramovitch A, Short T, Schweiger A (2021). The C Factor: cognitive dysfunction as a transdiagnostic dimension in psychopathology. Clin Psychol Rev.

[15] Bazanis E, Rogers RD, Dowson JH, Taylor P, Meux C, Staley C, Nevinson-Andres D, Taylor C, Robbins TW, Sahakin BJ (2002). Neurocognitive deficits in decision-making and planning of patients with DSM-III-R borderline personality disorder. Psychol Med.

[16] Paret C, Jennen-Steinmetz C, Schmahl C (2017). Disadvantageous decision-making in borderline personality disorder: partial support from a meta-analytic review. Neurosci Biobehav Rev.

[17] Rao S, Broadbear J (2019). Borderline personality disorder and depressive disorder. Australas Psychiatry.

[18] Ford JD, Courtois CA (2021). Complex PTSD and borderline personality disorder. Border Person. Dis. Emot. Dysregul..

[19] Unoka Z, Richman MJ (2016). Neuropsychological deficits in BPD patients and the moderator effects of co-occurring mental disorders: a meta-analysis. Clin Psychol Rev.

[20] Choate AM, Fatimah H, Bornovalova MA (2021). Comorbidity in borderline personality: understanding dynamics in development. Curr Opin Psychol.

[21] Moritz S, Schilling L, Wingenfeld K, Köther U, Wittekind C, Terfehr K, Spitzer C (2011). Psychotic-like cognitive biases in borderline personality disorder. J Behav Ther Exp Psychiatry.

[22] Puri P, Kumar D, Muralidharan K, Kishore MT (2018). Individuals with borderline personality disorder manifest cognitive biases implicated in psychosis. Psychiatry Res.

[23] McLean BF, Mattiske JK, Balzan RP (2017). Association of the jumping to conclusions and evidence integration biases with delusions in psychosis: a detailed meta-analysis. Schizophr Bull.

[24] Livet A, Navarri X, Potvin S, Conrod P (2020). Cognitive biases in individuals with psychotic-like experiences: a systematic review and a meta-analysis. Schizophr Res.

[25] Henquet C, van Os J, Pries LK, Rauschenberg C, Delespaul P, Kenis G, Luykx JJ, Lin BD, Richards AL, Akdede B (2020). A replication study of JTC bias, genetic liability for psychosis and delusional ideation. Psychol Med.

[26] Van Dael F, Versmissen D, Janssen I, Myin-Germeys I, Van Os J, Krabbendam L (2006). Data gathering: biased in psychosis?. Schizophr Bull.

[27] D’Agostino A, Monti MR, Starcevic V (2019). Psychotic symptoms in borderline personality disorder: an update. Curr Opin Psychiatry.

[28] West ML, Guest RM, Carmel A (2021). Comorbid early psychosis and borderline personality disorder: conceptualizing clinical overlap, etiology, and treatment. Personal Ment Health.

[29] Pearse LJ, Dibben C, Ziauddeen H, Denman C, McKenna PJ (2014). A study of psychotic symptoms in borderline personality disorder. J Nerv Ment Dis.

[30] Catalan A, Simons CJP, Bustamante S, Olazabal N, Ruiz E, De Artaza MG, Penas A, Maurottolo C, González A, Van Os J, Gonzalez-Torres MA (2015). Data gathering bias: trait vulnerability to psychotic symptoms?. PLoS ONE.

[31] Masland SR, Schnell SE, Shah TV (2020). Trust beliefs, biases, and behaviors in borderline personality disorder: empirical findings and relevance to epistemic trust. Curr Behav Neurosci Reports.

[32] Botsford J, Schulze L, Bohländer J, Renneberg B (2021). Interpersonal trust: development and validation of a self-report inventory and clinical application in patients with borderline personality disorder. J Pers Disord.

[33] Harvey N, Fischer I (1997). Taking advice: accepting help, improving judgment, and sharing responsibility. Organ Behav Hum Decis Process.

[34] Bonaccio S, Dalal RS (2006). Advice taking and decision-making: an integrative literature review, and implications for the organizational sciences. Organ Behav Hum Decis Process.

[35] Bailey PE, Leon T, Ebner NC, Moustafa AA, Weidemann G (2022). A meta-analysis of the weight of advice in decision-making. Curr Psychol.

[36] Kaliuzhna M, Chambon V, Franck N, Testud B, van der Henst JB (2012). Belief revision and delusions: how do patients with schizophrenia take advice?. PLoS ONE.

[37] Hofheinz C, Germar M, Schultze T, Michalak J, Mojzisch A (2017). Are depressed people more or less susceptible to informational social influence?. Cognit Ther Res.

[38] Scheunemann J, Gawęda Ł, Reininger KM, Jelinek L, Hildebrandt H, Moritz S (2020). Advice weighting as a novel measure for belief flexibility in people with psychotic-like experiences. Schizophr Res.

[39] Nicol K, Pope M, Sprengelmeyer R, Young AW, Hall J (2013). Social judgement in borderline personality disorder. PLoS ONE.

[40] Hepp J, Kieslich PJ, Schmitz M, Schmahl C, Niedtfeld I (2021). Negativity on two sides: individuals with borderline personality disorder form negative first impressions of others and are perceived negatively by them. Personal Disord Theory, Res Treat.

[41] Masland SR, Hooley JM (2020). When trust does not come easily: negative emotional information unduly influences trustworthiness appraisals for individuals with borderline personality features. J Pers Disord.

[42] Brook M, Hilty DM, Liu W, Hu R, Frye MA (2006). Discharge against medical advice from inpatient psychiatric treatment: a literature review. Psychiatr Serv.

[43] Kube T, Rozenkrantz L (2021). When beliefs face reality: an integrative review of belief updating in mental health and illness. Perspect Psychol Sci.

[44] Svaldi J, Philipsen A, Matthies S (2012). Risky decision-making in borderline personality disorder. Psychiatry Res.

[45] Linhartová P, Širůček J, Ejova A, Barteček R, Theiner P, Kašpárek T (2021). Dimensions of impulsivity in healthy people, patients with borderline personality disorder, and patients with attention-deficit/hyperactivity disorder. J Atten Disord.

[46] Renneberg B, Herm K, Hahn A, Staebler K, Lammers CH, Roepke S (2012). Perception of social participation in borderline personality disorder. Clin Psychol Psychother.

[47] Foxhall M, Hamilton-Giachritsis C, Button K (2019). The link between rejection sensitivity and borderline personality disorder: a systematic review and meta-analysis. Br J Clin Psychol.

[48] Cavicchioli M, Maffei C (2020). Rejection sensitivity in borderline personality disorder and the cognitive–affective personality system: a meta-analytic review. Personal Disord Theory, Res Treat.

[49] Sastre-Buades A, Ochoa S, Lorente-Rovira E, Barajas A, Grasa E, López-Carrilero R, Luengo A, Ruiz-Delgado I, Cid J, González-Higueras F, Sánchez-Alonso S, Baca-García E, Barrigón ML, Acevedo A, Anglès J, Argany MA, Barajas A, Barrigón ML, Beltrán M, Birulés I, Bogas JL, Camprubí N, Carbonero M, Carmona Farrés C, Carrasco E, Casañas R, Cid J, Conesa E, Corripio I, Cortes P, Crosas JM, de Apraiz A, Delgado M, Domínguez L, Escartí MJ, Escudero A, Esteban Pinos I, Figueras M, Franco C, García C, Gil V, Giménez-Díaz D, Gonzalez-Casares R, González Higueras F, González- Montoro ML, González E, Grasa Bello E, Guasp A, Huerta-Ramos ME, Huertas P, Jiménez-Díaz A, Lalucat LL, Llacer B, López-Alcayada R, López- Carrilero R, Lorente E, Luengo A, Mantecón N, Mas-Expósito L, Montes M, Moritz S, Murgui E, Nuñez M, Ochoa S, Palomer E, Paniego E, Peláez T, Pérez V, Planell K, Planellas C, Pleguezuelo-Garrote P, Pousa E, Rabella M, Renovell M, Rubio R, Ruiz- Delgado I, San Emeterio M, Sánchez E, Sanjuán J, Sans B, Schilling L, Sió H, Teixidó M, Torres P, Vila MA, Vila-Badia R, Villegas F, Villellas R (2021). Jumping to conclusions and suicidal behavior in depression and psychosis. J Psychiatr Res.

[50] Zanarini MC, Frankenburg FR, Dubo ED, Sickel AE, Trikha A, Levin A, Reynolds V (1998). Axis I comorbidity of borderline personality disorder. Am J Psychiatry.

[51] Schilling L, Wingenfeld K, Löwe B, Moritz S, Terfehr K, Köther U, Spitzer C (2012). Normal mind-reading capacity but higher response confidence in borderline personality disorder patients. Psychiatry Clin Neurosci.

[52] Schilling L, Wingenfeld K, Spitzer C, Nagel M, Moritz S (2013). False memories and memory confidence in borderline patients. J Behav Ther Exp Psychiatry.

[53] Scheunemann J, Fischer R, Moritz S (2021). Probing the hypersalience hypothesis—an adapted judge-advisor system tested in individuals with psychotic-like experiences. Front psychiatry.

[54] Henco L, Diaconescu AO, Lahnakoski JM, Brandi ML, Hörmann S, Hennings J, Hasan A, Papazova I, Strube W, Bolis D, Schilbach L, Mathys C (2020). Aberrant computational mechanisms of social learning and decision-making in schizophrenia and borderline personality disorder. PLoS Comput Biol.

[55] Fineberg SK, Leavitt J, Stahl DS, Kronemer S, Landry CD, Alexander-Bloch A, Hunt LT, Corlett PR (2018). Differential valuation and learning from social and nonsocial cues in borderline personality disorder. Biol Psychiatry.

[56] First MB, Gibbon M, Hilsenroth MJ, Segal DL (2004). The structured clinical interview for DSM-IV axis I disorders (SCID-I) and the structured clinical interview for DSM-IV axis II disorders (SCID-II). Comprehensive handbook of psychological assessment.

[57] Sheehan DV, Lecrubier Y, Sheehan KH, Amorim P, Janavs J, Weiller E, Hergueta T, Baker R, Dunbar GC (1998). The Mini-International Neuropsychiatric Interview (MINI): the development and validation of a structured diagnostic psychiatric interview for DSM-IV and ICD-10. J Clin Psychiatry.

[58] Vieira TF, Bottino A, Laurentini A, De Simone M (2014). Detecting siblings in image pairs. Vis Comput.

[59] Yaniv I, Milyavsky M (2007). Using advice from multiple sources to revise and improve judgments. Organ Behav Hum Decis Process.

[60] Soll JB, Larrick RP (2009). Strategies for revising judgment: how (and how well) people use others’ opinions. J Exp Psychol Learn Mem Cogn.

[61] Bohus M, Kleindienst N, Limberger MF, Stieglitz R-D, Domsalla M, Chapman AL, Steil R, Philipsen A, Wolf M (2009). The short version of the borderline symptom list (BSL-23): development and initial data on psychometric properties. Psychopathology.

[62] Beck A, Steer R, Brown G (1996). Manual for the beck depression inventory-II.

[63] Beck AT, Baruch E, Balter JM, Steer RA, Warman DM (2004). A new instrument for measuring insight: the beck cognitive insight scale. Schizophr Res.

[64] Kliem S, Lohmann A, Mößle T, Brähler E (2017). German beck scale for suicide ideation (BSS): psychometric properties from a representative population survey. BMC Psychiatry.

[65] Hamilton M (1960). A rating scale for depression. J Neurol Neurosurg Psychiatry.

[66] Baron RM, Kenny DA (1986). The moderator–mediator variable distinction in social psychological research: conceptual, strategic, and statistical considerations. J Pers Soc Psychol.

[67] RStudio Team (2015) RStudio: integrated development environment for R

[68] R Core Team (2014) R: a language and environment for statistical computing

[69] Moritz S, Woodward TS (2005). Jumping to conclusions in delusional and non-delusional schizophrenic patients. Br J Clin Psychol.

[70] Moritz S, Scheunemann J, Lüdtke T, Westermann S, Pfuhl G, Balzan RP, Andreou C (2020). Prolonged rather than hasty decision-making in schizophrenia using the box task Must we rethink the jumping to conclusions account of paranoia?. Schizophr Res.

[71] Tripoli G, Quattrone D, Ferraro L, Gayer-Anderson C, Rodriguez V, La Cascia C, La Barbera D, Sartorio C, Seminerio F, Tarricone I (2020). Jumping to conclusions, general intelligence, and psychosis liability: findings from the multi-centre EU-GEI case-control study. Psychol Med.

[72] Niedtfeld I (2017). Experimental investigation of cognitive and affective empathy in borderline personality disorder: effects of ambiguity in multimodal social information processing. Psychiatry Res.

[73] Thome J, Liebke L, Bungert M, Schmahl C, Domes G, Bohus M, Lis S (2016). Confidence in facial emotion recognition in borderline personality disorder. Personal Disord Theory Res Treat.

[74] Winter D, Bohus M, Lis S (2017). Understanding negative self-evaluations in borderline personality disorder—a review of self-related cognitions, emotions, and motives. Curr Psychiatry Rep.

[75] Gino F, Brooks AW, Schweitzer ME (2012). Anxiety, advice, and the ability to discern: feeling anxious motivates individuals to seek and use advice. J Pers Soc Psychol.

[76] Nicol K, Pope M, Sprengelmeyer R, Young AW, Hall J (2013). Social judgement in borderline personality disorder. PLoS ONE.

[77] Richetin J, Poggi A, Ricciardelli P, Fertuck EA, Preti E (2018). The emotional components of rejection sensitivity as a mediator between borderline personality disorder and biased appraisal of trust in faces. Clin Neuropsychiatry.

[78] Preuss N, Brändle LS, Hager OM, Haynes M, Fischbacher U, Hasler G (2016). Inconsistency and social decision making in patients with borderline personality disorder. Psychiatry Res.

[79] Poggi A, Richetin J, Preti E (2019). Trust and rejection sensitivity in personality disorders. Curr Psychiatry Rep.

[80] Brüne M (2016). Borderline personality disorder. Evol Med Public Heal.

[81] Moritz S, Woodward TS (2007). Metacognitive training in schizophrenia: from basic research to knowledge translation and intervention. Curr Opin Psychiatry.

[82] Nosek BA, Ebersole CR, DeHaven AC, Mellor DT (2018). The preregistration revolution. Proc Natl Acad Sci.

[83] Andreou C, Moritz S, Veith K, Veckenstedt R, Naber D (2013). Dopaminergic modulation of probabilistic reasoning and overconfidence in errors: a double-blind study. Schizophr Bull.

[84] Andreou C, Schneider BC, Braun V, Kolbeck K, Gallinat J, Moritz S (2015). Dopamine effects on evidence gathering and integration. J Psychiatry Neurosci.

[85] Baker SC, Konova AB, Daw ND, Horga G (2019). A distinct inferential mechanism for delusions in schizophrenia. Brain.

