# Supplementary material for: Can you trust this source? Advice taking in borderline personality disorder
Source: Eur Arch Psychiatry Clin Neurosci. 2023 Jan 11;273(4):875–85. doi: 10.1007/s00406-022-01539-w (PMC10238350; doi:10.1007/s00406-022-01539-w)
Supplement: Supplementary file 1 — Supplementary file1 (DOCX 21 KB) [file 406_2022_1539_MOESM1_ESM.docx]

**Post-Hoc Analysis of Main Outcomes**

As the distance of advice (difference between advice and initial estimate) affects advice taking [1], the four trials per task type consisted of different distances of advice. However, none of the eight trials evoked significant group differences in either outcome (NoRfA *p* ≥ .237, *d* ≤ 0.29; Confidence *p* ≥ .194, *d* ≤ 0.32; RAAW *p* ≥ .103, *d* ≤ 0.38).

In our preregistered protocol, we only specified outliers for RAAW (truncated to 1 or 0; see above). As random outliers might have contributed to an incorrect non-rejection of the null hypothesis (beta error; [2]), we searched for outliers on our pooled main outcomes post-hoc using a liberal threshold of two median absolute deviations (MAD; [3]). This led to an exclusion of 12 observations for NoRfA (median = 0.25; *MAD* = 0.37), 16 observations for Confidence (median = 2.75; *MAD* = 0.37), and 3 observations for RAAW (median = 0.14; *MAD* = 0.13). However, even after exclusion, effect sizes were still small (*d* ≤ 0.21) and results remained non-significant (*p* ≥ .432) for the pooled scores. Hence, there is no indication that random outliers caused a beta error (non-significant group differences despite an effect).

Confidence was measured on a Likert scale. There is a debate on whether metric tests should be used for analyzing Likert scales [4, 5] as we have predefined our analysis in the pre-registration protocol. Therefore, we additionally provide post-hoc analyses using the non-parametric Mann-Whitney-Test for the median Confidence scores: Patients with BPD had lower median Confidence scores than HCs (Pooled score: Median(BPD) = 2.625, Median (HC) = 2.750; Age Task: Median (BPD) = 2.500, Median (HC) = 2.75; Hostility Task: Median (BPD) = 2.750, Median (HC) = 2.750), replicating the comparison of the arithmetic means. The nonparametric Mann-Whitney-Tests revealed no group differences for any Confidence subscore (Pooled score: *U* = 498.000, *z* = 0.895, *p* = .371; Age Task: *U* = 488.500, *z* = 1.029, *p* = .303; Hostility Task: *U* = 515.500, *z* = 0.686, *p* = .493).

**Preregistered Mediation Analysis**

While self-esteem (RSES) correlated with RAAW with *r* = −.31, this correlation was not significant (*p* = .057, *n* = 38). RSES did not correlate with NoRfA (*r* = −.11, *p* = .514, *n* = 38) or Confidence (*r* = −.00, *p* = .989, *n* = 38). And, as described in the main article, symptom severity did not correlate with JAS outcome measures. Consequently, and in line with our preregistered protocol, there was no indication of a mediation by self-esteem on the relationship between symptom severity and JAS outcome measures, additionally indicated by corresponding Sobel tests (RAAW: *p* = .172, NoRfA: *p* = .441, Confidence: *p* = .929; all *n* = 38).

**Correlations Between Outcomes**

In the entire sample (*n* = 68), there was a positive correlation between higher NoRfA and increased RAAW scores (*r* = .46, p < .001). NoRfA did not correlate with Confidence (*r* = -.11, *p* = .369); Confidence did not correlate with RAAW (*r* = −.07, *p* = .594).

**References**

1. Schultze T, Rakotoarisoa A-F, Schulz-Hardt S (2015) Effects of distance between initial estimates and advice on advice utilization. Judgm Decis Mak 10:

2. Leys C, Delacre M, Mora YL, Lakens D, Ley C (2019) How to classify, detect, and manage univariate and multivariate outliers, with emphasis on pre-registration. Int Rev Soc Psychol 32:. https://doi.org/10.5334/irsp.289

3. Leys C, Ley C, Klein O, Bernard P, Licata L (2013) Detecting outliers: Do not use standard deviation around the mean, use absolute deviation around the median. J Exp Soc Psychol 49:764–766. https://doi.org/10.1016/j.jesp.2013.03.013

4. Carifio J, Perla RJ (2007) Ten Common Misunderstandings, Misconceptions, Persistent Myths and Urban Legends about Likert Scales and Likert Response Formats and their Antidotes. J Soc Sci 3:. https://doi.org/10.3844/jssp.2007.106.116

5. Mircioiu C, Atkinson J (2017) A Comparison of Parametric and Non-Parametric Methods Applied to a Likert Scale. Pharmacy 5:. https://doi.org/10.3390/pharmacy5020026
