# Supplementary material for: Can you trust this source? Advice taking in borderline personality disorder
Source: Eur Arch Psychiatry Clin Neurosci. 2023 Jan 11;273(4):875–85. doi: 10.1007/s00406-022-01539-w (PMC10238350; doi:10.1007/s00406-022-01539-w)
Supplement: Supplementary file 2 — Supplementary file2 (DOCX 13 KB) [file 406_2022_1539_MOESM2_ESM.docx]

| **Table S1** |  |  |
| --- | --- | --- |
| *Frequency of comorbidities based on the Mini-International Neuropsychiatric Interview (M.I.N.I.) and duration of illness for the patient group (*n *= 38).* | | |
| Any depressive disorder | *n* = 34 | 89.5% |
| Current depression | *n* = 28 | 73.7% |
| Lifetime manic or hypomanic episode | *n* = 5 | 13.2% |
| Any anxiety disorder | *n* = 28 | 73.7% |
| Obsessive–compulsive disorder | *n* = 3 | 7.9% |
| Any alcohol-use disorder | *n* = 21 | 55.3% |
| Any substance-use disorder (non-alcohol) | *n* = 15 | 39.5% |
| Any eating disorder | *n* = 1 | 2.6% |
| Mean number of comorbidities in addition to BPD diagnosis | *M* = 3.5 | *SD* = 1.4 |
| Duration of illness (in months)^a^ | *M* = 120.5 | *SD* = 77.6 |
| Number of hospital stays^a^ | *M* = 4.8 | *SD* = 4.1 |
| *Note.* For the M.I.N.I., the subsection on traumatic disorder was not assessed. ^a^ Based on interview data. | | |
